# ﻿Novel *Helicosporium* and *Neohelicomyces* (Tubeufiaceae, Tubeufiales) species from terrestrial habitats in China and Thailand

**DOI:** 10.3897/mycokeys.112.140211

**Published:** 2025-01-10

**Authors:** Tao Peng, Yong-Zhong Lu, Song Bai, Jing-Yi Zhang, Xing-Juan Xiao, Na Wu, Jian Ma

**Affiliations:** 1 Department of Brewing Engineering, Moutai Institute, Renhuai 564500, China; 2 School of Food and Pharmaceutical Engineering, Guizhou Institute of Technology, Guiyang 550003, China; 3 Guizhou Industry Polytechnic College, Guiyang 550008, China; 4 Center of Excellence in Fungal Research, Mae Fah Luang University, Chiang Rai 57100, Thailan; 5 School of Science, Mae Fah Luang University, Chiang Rai, 57100, Thailand; 6 School of Life Science and Technology, Center for Informational Biology, University of Electronic Science and Technology of China, Chengdu 611731, China

**Keywords:** Helicosporous fungi, phylogeny, taxonomy, terrestrial habitats

## Abstract

During our investigations of saprobic fungi, five fungal collections from terrestrial habitats in China and Thailand were examined using both morphological and multi-gene phylogenetic approaches (LSU, ITS, *tef*1-α, and *rpb*2), resulting in the identification of three novel species: *Helicosporiumrubrum*, *Neohelicomycesmaolanensis*, and *N.subtropicus*. *Helicosporium* and *Neohelicomyces* are morphologically similar in their asexual morphs but can be distinguished based on their molecular phylogenetic data. In this study, our new species, *Helicosporiumrubrum*, represents the fourth sexual species within the genus, characterized by yellow-brown ascomata and fusiform ascospores. Detailed descriptions, illustrations, phylogenetic analysis results, and corresponding notes are provided to clarify the distinctions between these new species and related taxa.

## ﻿Introduction

*Helicosporium* was introduced by Nees (1817), with *H.vegetum* as the type species. Currently, 108 species are listed in Index Fungorum (2024); however, based on morphological data comparison and molecular data, only 28 are accepted within the genus *Helicosporium* (Lu et al. 2018b; Xiao et al. 2023; Ma et al. 2024b). Of these, 27 species have been found in freshwater and terrestrial habitats in various regions, including Austria, Belgium, Britain, Canada, China, Cuba, India, Thailand, and the USA (Lu et al. 2017a, 2018b, 2022; Dong et al. 2020; Boonmee et al. 2021; Hsieh et al. 2021; Xiao et al. 2023; Ma et al. 2024b). The remaining species, *H.vegetum*, has been found widely distributed in terrestrial habitats globally (Nees 1817; Linder 1929; Samuels and Muller 1979; Barr 1980; Goos 1989; Morgan-Jones and Goos 1992; Spatafora et al. 2006; Tsui et al. 2006; Zhao et al. 2007; Boonmee et al. 2014; Lu et al. 2018b; Ma et al. 2024b). So far, only *H.flavum*, *H.sexuale*, and *H.vegetum* have been described as sexual morphs in this genus, all of which have been confirmed through DNA sequence data (Boonmee et al. 2014, 2021; Brahmanage et al. 2017). These sexual morphs are characterized by solitary, yellowish-brown to greenish ascomata, cylindric-clavate, eight-spored bitunicate asci, and hyaline to yellowish-brown elongate-fusiform ascospores (Ma et al. 2024b). In contrast, their asexual morphs form pale yellow to yellow-green colonies on natural woody substrates, with erect, setiferous conidiophores and helicoid, hyaline to yellow-green conidia (Xiao et al. 2023; Ma et al. 2024b).

*Neohelicomyces* was established by Luo et al. (2017), with *N.aquaticus* as the type species, based on molecular and morphological data. The genus now includes 25 species; all are asexual morphs supported by molecular evidence. No sexual stages have been reported for this genus (Ma et al. 2024a, 2024b). *Neohelicomyces* species occupy both freshwater and terrestrial habitats and have a worldwide distribution and have been reported from China, Czech Republic, Germany, Italy, Japan, Netherlands, Thailand, and the USA (Luo et al. 2017; Lu et al. 2018b, 2022; Crous et al. 2019a, 2019b; Dong et al. 2020; Hsieh et al. 2021; Yang et al. 2023; Ma et al. 2024a, 2024b). *Neohelicomyces* species are characterized by macronematous, mononematous, erect, septate conidiophores with holoblastic conidiogenous cells and tightly or loosely coiled helicoid conidia (Hsieh et al. 2021; Lu et al. 2022; Yang et al. 2023; Ma et al. 2024a, 2024b).

Previous studies have highlighted the potential of *Helicosporium* and *Neohelicomyces* species to produce secondary metabolites with bioactive properties (Kim et al. 2003; Lee et al. 2006, 2013; Choi et al. 2012; Zheng et al. 2023). For example, *Helicosporiumnizamabadense* has demonstrated inhibitory effects against several agricultural pathogenic fungi, including *Botrytiscinerea*, *Fusariumoxysporum*, *Phytophthoradrechsleri*, and *Rhizoctoniasolani* (Kim et al. 2003; Lee et al. 2006, 2013). Additionally, 2-methylresorcinol, isolated from *Helicosporium* sp., exhibited antimicrobial activities against bacteria and fungi (Choi et al. 2012). More recently, two alkaloid compounds from *Neohelicomyceshyalosporus* were shown to have moderate cytotoxic effects on human cancer cells (Zheng et al. 2023). Therefore, the metabolites of *Helicosporium* and *Neohelicomyces* species could serve as promising sources for developing drugs to prevent and manage human tumors.

This study collected five saprophytic fungi from terrestrial habitats in Chiang Mai, Thailand, and Guizhou Province, China. Based on detailed morphological comparison and multigene phylogenetic analyses (LSU, ITS, *tef*1-α, and *rpb*2), three novel species, *viz. Helicosporiumrubrum*, *Neohelicomycesmaolanensis*, and *N.subtropicus*, were isolated and identified.

## ﻿Materials and methods

### ﻿Sample collection, examination, and isolation

Fresh specimens were collected from terrestrial habitats in China and Thailand between September 2020 and April 2022, and important collection information *in situ* was noted as per Rathnayaka et al. (2024). Fungal colonies and micro-morphological structures on the surface of natural substrates were observed using a stereomicroscope (SMZ-168, Nikon, Japan) and photographed with an ECLIPSE Ni compound microscope (Nikon, Tokyo, Japan) equipped with a Canon 90D digital camera.

Single spore isolations were performed following the method outlined by Chomnunti et al. (2014). Germinating spores were aseptically transferred to fresh potato dextrose agar (PDA) plates, according to the method described by Senanayake et al. (2020). Colony characteristics on PDA, such as shape, color, size, margin, and elevation, were monitored and recorded.

### ﻿Material deposition

Dried materials were deposited in the Herbarium of Mae Fah Luang University (Herb. MFLU), Chiang Rai, Thailand, and the Herbarium of Guizhou Academy of Agriculture Sciences (Herb. GZAAS), Guiyang, China. Cultures were deposited at Mae Fah Luang University Culture Collection (MFLUCC), Chiang Rai, Thailand, and the Guizhou Culture Collection (GZCC), Guiyang, China. The Faces of Fungi numbers were obtained following the guidelines outlined by Jayasiri et al. (2015). The newly introduced taxa were registered in the MycoBank database (https://www.mycobank.org/).

### ﻿DNA extraction, PCR amplification, and sequencing

Fresh mycelia were scraped with a sterilized surgical knife and transferred to a sterilized 1.5 mL microcentrifuge tube. Genomic DNA was extracted using the Biospin Fungus Genomic DNA Extraction Kit (BioFlux, China), following the manufacturer’s protocol. LSU, ITS, *tef*1-α, and *rpb*2 sequence fragments were amplified using primer pairs LR0R/LR5 (Vilgalys and Hester 1990), ITS5/ITS4 (White et al. 1990), EF1-983F/EF1-2218R (Rehner and Buckley 2005), and fRPB2-5F/fRPB2-7cR (Liu et al. 1999), respectively. The PCR amplification reactions were conducted in a 50 µL reaction volume, consisting of 44 µL of 1.1 × T3 Super PCR Mix (Qingke Biotech, Chongqing, China), 2 µL each of forward and reverse primers, and 2 µL of DNA. The thermal cycling parameters for LSU, ITS, *tef*1-α, and *rpb*2 regions followed the method described by Ma et al. (2024a). The PCR products were analyzed using 1% agarose gel electrophoresis, and the sequencing results were obtained by Beijing Qingke Biotechnology Co., Ltd.

### ﻿Phylogenetic analyses

Newly generated DNA sequence data for each region were checked using BioEdit v 7.0.5.3 (Hall 1999) and assembled with SeqMan v. 7.0.0 (DNASTAR, Madison, WI, USA; Swindell and Plasterer 1997). Classification of the newly introduced taxa was analyzed using the BLASTn tool in GenBank (https://www.ncbi.nlm.nih.gov/). Sequences used in this study were downloaded from GenBank (Table 1). Single-gene datasets were aligned using MAFFT v.7.473 (https://mafft.cbrc.jp/alignment/server/, Katoh et al. 2019) and subsequently trimmed with trimAl.v1.2rev59 software (Capella-Gutiérrez et al. 2009). The trimmed datasets were concatenated (LSU-ITS-*tef*1-α-*rpb*2) using SequenceMatrix-Windows-1.7.8 software (Vaidya et al. 2011). The maximum likelihood (ML) tree was constructed using the IQ-TREE web server (http://iqtree.cibiv.univie.ac.at/, Nguyen et al. 2015; Zeng et al. 2023). Bayesian Inference (BI) was conducted in accordance with the methods described by Ma et al. (2022). The trimmed Fasta file for each gene dataset was converted to Nexus format for Bayesian analysis using AliView v. 1.27 (Daniel et al. 2010). The best-fit substitution model for the LSU-ITS-*tef*1-α-*rpb*2 matrices was selected using MrModeltest 2.3, based on the Akaike Information Criterion (AIC) (Nylander et al. 2008).

**Table 1.** Taxa used in this study and their GenBank accession numbers.

Taxon	Strain	GenBank Accessions			
		LSU	ITS	*tef*1-α	*rpb*2
*Acanthostigmachiangmaiensis*	MFLUCC 10-0125^T^	JN865197	JN865209	KF301560	-
*Acanthostigmaperpusillum*	UAMH 7237	AY856892	AY916492	-	-
*Botryosphaeriaagaves*	MFLUCC 10-0051	JX646807	JX646790	-	-
*Botryosphaeriadothidea*	CBS 115476	DQ678051	KF766151	DQ767637	DQ677944
*Helicosporiumacropleurogenum*	CGMCC 3.25563^T^	PP639430	PP626574	PP596333	PP596460
*Helicosporiumaquaticum*	GZCC 22-2120	PP639431	PP626575	PP596334	PP596461
*Helicosporiumaquaticum*	MFLUCC 17-2008^T^	MH558859	MH558733	MH550924	MH551049
*Helicosporiumbrunneisporum*	CGMCC 3.2554^2^T	PP639433	PP626577	PP596336	PP596463
*Helicosporiumchangjiangense*	GZCC 22-2113^T^	PP639434	PP626578	PP596337	PP596464
*Helicosporiumflavisporum*	MFLUCC 17-2020^T^	MH558860	MH558734	MH550925	MH551050
*Helicosporiumflavum*	GZCC 23-0487	PP639435	PP626579	PP596338	PP596465
*Helicosporiumflavum*	MFLUCC 16-1230^T^	KY873621	KY873626	KY873285	-
*Helicosporiumhainanense*	GZAAS 22-2006^T^	OP508770	OP508730	OP698081	OP698070
*Helicosporiumjiangkouense*	HKAS 128933^T^	PP639436	PP626580	PP596339	PP596466
*Helicosporiumjiangkouense*	HKAS 128901	-	PP626581	-	-
*Helicosporiumlatisporum*	HKAS 128960^T^	PP639437	PP626582	PP596340	PP596467
*Helicosporiumliuzhouense*	GZCC 22-2014^T^	OQ981402	OQ981394	OQ980476	OQ980474
*Helicosporiumluteosporum*	MFLUCC 16-0226^T^	KY321327	KY321324	KY792601	MH551056
*Helicosporiumluteosporum*	MFLUCC 16-1233	KY873624	-	-	-
*Helicosporiummultidentatum*	GZCC 22-2013^T^	OQ981403	OQ981395	OQ980477	OQ980475
*Helicosporiumnanningense*	GZCC 22-2175^T^	OR066425	OR066418	OR058864	OR058857
*Helicosporiumramosiphorum*	CGMCC 3.25541^T^	PP639432	PP626576	PP596335	PP596462
***Helicosporiumrubrum***	**MFLUCC 24-0090^T^**	**PQ098514**	**PQ098477**	**PQ490681**	**PQ490675**
***Helicosporiumrubrum***	**GZCC 24-0149**	**PQ522499**	**PQ522497**	**PQ490680**	**PQ490674**
*Helicosporiumsetiferum*	GZCC 23-0152	PP639438	PP626583	PP596341	PP596468
*Helicosporiumsetiferum*	BCC 3332	AY856907	AY916490	-	-
*Helicosporiumsetiferum*	BCC 8125	-	AY916491	-	-
*Helicosporiumsetiferum*	MFLUCC 17-1994^T^	MH558861	MH558735	MH550926	MH551051
*Helicosporiumsetiferum*	MFLUCC 17-2006	MH558862	MH558736	MH550927	MH551052
*Helicosporiumsetiferum*	MFLUCC 17-2007	MH558863	MH558737	MH550928	MH551053
*Helicosporiumsexuale*	GZCC 22-2007	OP508771	OP508731	OP698082	OP698071
*Helicosporiumsexuale*	MFLUCC 16-1244^T^	MZ538537	MZ538503	MZ567082	MZ567111
*Helicosporium* sp.	NBRC 9014	AY856903	AY916489	-	-
*Helicosporiumthailandense*	MFLUCC 18-1407^T^	MN913718	MT627698	MT954371	-
*Helicosporiumvegetum*	GZCC 23-0060	PP639439	PP626584	PP596342	PP596469
*Helicosporiumvegetum*	CBS 941.72	AY856883	AY916488	-	-
*Helicosporiumvegetum*	NBRC 30345	AY856896	-	-	-
*Helicosporiumvegetum*	CBS 254.75	DQ470982	-	DQ471105	-
*Helicosporiumvegetum*	CBS 269.52	AY856893	AY916487	-	-
*Helicosporiumvesicarium*	MFLUCC 17-1795^T^	MH558864	MH558739	MH550930	MH551055
*Helicosporiumviridiflavum*	MFLUCC 17-2336^T^	-	MH558738	MH550929	MH551054
*Helicosporiumviridisporum*	GZCC 23-0044	PP639440	PP626585	-	-
*Helicosporiumviridisporum*	GZCC 23-0045	PP639441	PP626586	PP596343	PP596470
*Helicosporiumviridisporum*	GZCC 22-2008^T^	OP508776	OP508736	OP698087	OP698076
*Helicotubeufiahydei*	MFLUCC 17-1980^T^	MH290026	MH290021	MH290031	MH290036
*Helicotubeufiajonesii*	MFLUCC 17-0043^T^	MH290025	MH290020	MH290030	MH290035
*Muripulchraaquatica*	KUMCC 15-0245	KY320550	KY320533	KY320563	MH551057
*Muripulchraaquatica*	KUMCC 15-0276	KY320551	KY320534	KY320564	MH551058
*Muripulchraaquatica*	DLUCC 0571	KY320548	KY320531	-	-
*Muripulchraaquatica*	MFLUCC 15-0249^T^	KY320549	KY320532	-	-
*Neohelicomycesacropleurogenus*	CGMCC 3.25549^T^	PP639450	PP626594	PP596351	PP596478
*Neohelicomycesaquaticus*	KUMCC 15-0463	KY320546	KY320529	KY320562	MH551065
*Neohelicomycesaquaticus*	MFLUCC 16-0993^T^	KY320545	KY320528	KY320561	MH551066
*Neohelicomycesaseptatus*	CGMCC 3.25564^T^	PP639451	PP626595	PP596352	PP596479
*Neohelicomycesdehongensis*	MFLUCC 18-1029^T^	MN913709	NR_171880	MT954393	-
*Neohelicomycesdenticulatus*	GZCC 19-0444^T^	MW133855	OP377832	-	-
*Neohelicomycesdenticulatus*	GZCC 23-0073^T^	PP639452	PP626596	PP596353	PP596480
*Neohelicomycesdeschampsiae*	CPC 33686^T^	MK442538	MK442602	-	-
*Neohelicomycesedgeworthiae*	CGMCC 3.25565^T^	PP639453	PP626597	PP596354	PP596481
*Neohelicomycesgrandisporus*	KUMCC 15-0470^T^	KX454174	KX454173	-	MH551067
*Neohelicomycesguizhouensis*	GZCC 23-0725^T^	PP512973	PP512969	PP526727	PP526733
*Neohelicomycesguizhouensis*	GZCC 23-0726	PP512974	PP512970	PP526728	PP526734
*Neohelicomycesguttulatus*	CGMCC 3.25550^T^	PP639454	PP626598	PP596355	-
*Neohelicomycesguttulatus*	GZCC 23-0406	PP639455	PP626599	PP596356	PP596482
*Neohelicomyceshainanensis*	GZCC 22-2009^T^	OP508774	OP508734	OP698085	OP698074
*Neohelicomyceshainanensis*	GZCC 22-2027	OP508775	OP508735	OP698086	OP698075
*Neohelicomyceshelicosporus*	GZCC 23-0633^T^	PP512975	PP512971	PP526729	PP526735
*Neohelicomyceshelicosporus*	GZCC 23-0634	PP512976	PP512972	PP526730	PP526736
*Neohelicomyceshyalosporus*	GZCC 16-0086^T^	MH558870	MH558745	MH550936	MH551064
*Neohelicomyceshydei*	GZCC 23-0727^T^	PP512977	-	PP526731	PP526737
*Neohelicomyceshydei*	GZCC 23-0728	PP512978	-	PP526732	PP526738
*Neohelicomyceslignicola*	CGMCC 3.25551^T^	PP639456	PP626600	PP596357	PP596483
*Neohelicomyceslongisetosus*	NCYU-106H1-1-1^T^	-	MT939303	-	-
*Neohelicomycesmacrosporus*	CGMCC 3.25552^T^	PP639457	PP626601	PP596358	PP596484
***Neohelicomycesmaolanensis***	**GZCC 23-0079^T^**	**PQ098529**	-	**PQ490683**	**PQ490677**
***Neohelicomycesmaolanensis***	**GZCC 24-0148**	**PQ522500**	-	**PQ490682**	**PQ490676**
*Neohelicomycesmelaleucae*	CPC 38042^T^	MN567661	MN562154	MN556835	-
*Neohelicomycesmelaleucae*	KUNCC 23-14314	PP664112	PP664108	PP680211	-
*Neohelicomycespallidus*	CBS 245.49	-	MH856510	-	-
*Neohelicomycespallidus*	CBS 271.52	AY856887	AY916461	-	-
*Neohelicomycespallidus*	CBS 962.69	AY856886	AY916460	-	-
*Neohelicomycesdenticulatus*	UAMH 10535	AY856913	AY916462	-	-
*Neohelicomycespandanicola*	KUMCC 16-0143^T^	MH260307	MH275073	MH412779	-
*Neohelicomycesqixingyaensis*	CGMCC 3.25569^T^	PP639458	PP626602	PP596359	PP596485
*Neohelicomycessubmersus*	MFLUCC 16-1106^T^	KY320547	KY320530	-	MH551068
***Neohelicomycessubtropicus***	**GZCC 23-0076^T^**	**PQ098530**	**PQ098492**	**PQ490685**	**PQ490679**
***Neohelicomycessubtropicus***	**GZCC 24-0147**	**PQ522501**	**PQ522498**	**PQ490684**	**PQ490678**
*Neohelicomycesthailandicus*	MFLUCC 11-0005^T^	MN913696	NR_171882	-	
*Neohelicomycesthailandicus*	GZCC 23-0400	PP639459	PP626603	PP596360	PP596486
*Neohelicomycesxiayadongensis*	CGMCC 3.25568^T^	PP639460	PP626604	PP596361	PP596487
*Neohelicomycesyunnanensis*	GZCC 23-0735^T^	PP664113	PP664109	–	–
Tubeufiaceae sp.	ATCC 42524	AY856911	AY916458	-	-
*Tubeufiaguttulata*	GZCC 23-0404^T^	OR030834	OR030841	OR046678	OR046684
*Tubeufiahainanensis*	GZCC 22-2015^T^	OR030835	OR030842	OR046679	OR046685
*Neohelicomycesxiayadongensis*	MUCL 15702	AY856873	AY916459	-	-
*Tubeufiajavanica*	MFLUCC 12-0545^T^	KJ880036	KJ880034	KJ880037	-
*Tubeufiakrabiensis*	MFLUCC 16-0228^T^	MH558917	MH558792	MH550985	MH551118
*Tubeufialatispora*	MFLUCC 16-0027^T^	KY092412	KY092417	KY117033	MH551119
*Tubeufialaxispora*	MFLUCC 16-0232^T^	KY092408	KY092413	KY117029	MF535287
*Tubeufiamachaerinae*	MFLUCC 17-0055	MH558920	MH558795	MH550988	MH551122
*Tubeufiamackenziei*	MFLUCC 16-0222^T^	KY092410	KY092415	KY117031	MF535288
*Tubeufiamuriformis*	GZCC 22-2039^T^	OR030836	OR030843	OR046680	OR046686
*Tubeufianigroseptum*	CGMCC 3.20430^T^	MZ853187	MZ092716	OM022002	OM022001
*Tubeufiapandanicola*	MFLUCC 16-0321^T^	MH260325	MH275091	-	-

Note: “^T^” indicates ex-type strains. The newly generated sequences are highlighted in bold black. “-” indicates the unavailable data in GenBank.

The ML and BI trees were visualized using FigTree v. 1.4.4 and edited with Adobe Illustrator CC 2019 (v. 23.1.0; Adobe Systems, USA). Photoplates were created using Adobe Photoshop CC 2019 (Adobe Systems, USA) and the Tarosoft (R) Image Frame Work program.

### ﻿Phylogenetic results

**Figure 1.**
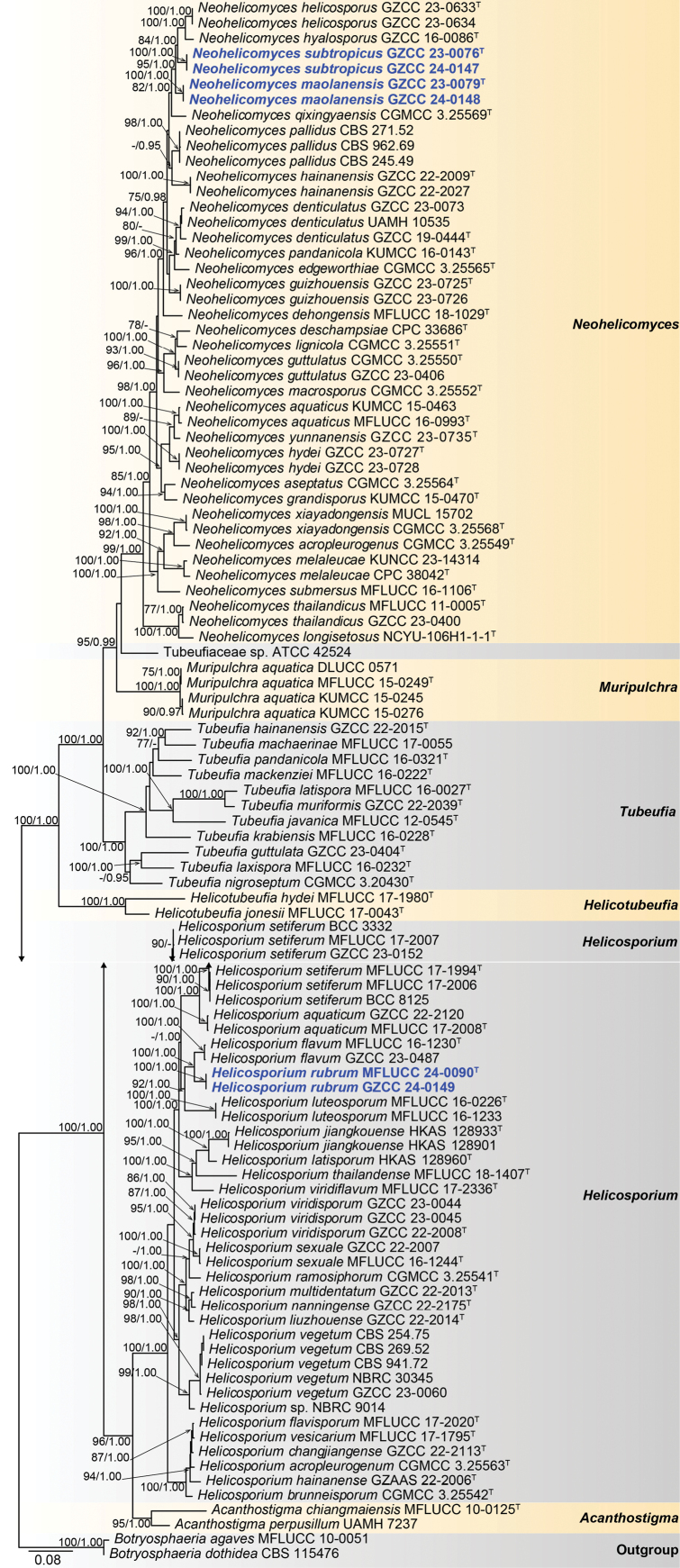
Phylogram generated from the best scoring of the RAxML tree based on the combined LSU, ITS, *tef*1-α, and *rpb*2 sequence dataset, indicating *Helicosporium* and *Neohelicomyces* species. *Botryosphaeriaagaves* (MFLUCC 10–0051) and *B.dothidea* (CBS 115476) were selected as outgroup taxa. Bootstrap support values from maximum likelihood (ML) equal to or greater than 75% and Bayesian posterior probabilities (PP) equal to or greater than 0.95 are shown near the nodes as ML/PP, respectively. “^T^” denotes ex-holotype strains. New species are in bold blue.

The phylogenetic positions of the newly introduced species were elucidated through a multi-gene phylogenetic analysis incorporating LSU, ITS, *tef*1-α, and *rpb*2 sequences. The concatenated sequence matrix consisted of 3,418 characters: LSU (1–851), ITS (852–1,444), *tef*1-α (1,445–2,356), and *rpb*2 (2,357–3,418), encompassing 102 ingroup taxa and two outgroup taxa, *Botryosphaeriaagaves* and *B.dothidea*. Both ML and BI analyses yielded similar tree topologies. Fig. 1 presents the highest-scoring ML tree, which achieved a final likelihood value of -31,594.957.

Based on the phylogenetic analysis (Fig. 1), our collections belong to *Helicosporium* and *Neohelicomyces* within Tubeufiaceae (Tubeufiales, Dothideomycetes). Two isolates (MFLUCC 24–0090 and GZCC 24–0149) formed a sister clade with *Helicosporiumflavum* (MFLUCC 16–1230 and GZCC 23–0487), supported by 100% ML and 1.00 PP. Additionally, our new isolates (GZCC 23–0079 and GZCC 24–0148) cluster together with a clade comprising *N.helicosporus*, *N.hyalosporus*, *N.qixingyaensis*, and *N.subtropicus*. Furthermore, GZCC 23–0076 and GZCC 24–0147 grouped together and clustered sister to *N.helicosporus* (GZCC 23–0633 and GZCC 23–0634) and *N.hyalosporous* (GZCC 16–0086), supported by 84% ML and 1.00 PP.

#### ﻿ Helicosporium rubrum

Taxon classificationFungiTubeufialesTubeufiaceae

J. Ma & Y.Z. Lu sp. nov.

9D454E4C-7782-5D83-B8BD-8046B7EB7022

902923

Facesoffungi Number: FoF16908

Fig. 2

##### Holotype.

MFLU 24–0035.

##### Etymology.

‘‘*rubrum*’’ refers to the red-brown colonies on the woody substrate.

##### Description.

***Saprobic*** on decaying wood in a terrestrial habitat. Asexual morph Undetermined. ***Sexual morph*: *Ascomata*** 151–185.5 µm high, 138–157 µm diam., superficial, seated on a subiculum, solitary, scattered, globose to subglobose, bright reddish yellow to brown yellow, with central narrow ostiole; setae were not observed, comprising short projections of setae-like, 10–35 × 4.5–8 µm. ***Peridium*** 17.5–22 µm wide, composed of several layers of hyaline to bright yellow cells of ***textura angularis***, outer layer yellow cells, and inner layer pale yellow to hyaline cells. ***Hamathecium*** comprising numerous, 1.5–2.5 µm wide, filiform, branched, septate, hyaline pseudoparaphyses. ***Asci*** 51–77 × 8–12.5 µm (x̄ = 64 × 10 μm, n = 20), 8-spored, bitunicate, fissitunicate, cylindrical to clavate or saccate, short-pedicellate, apically rounded, basally flexious. ***Ascospores*** 27–35 × 3–4.5 µm (x̄ = 31.5 × 4 μm, n = 20), overlapping 2–3-seriate, fusiform, tapering towards the ends, widest at the central part, straight to slightly curved, multi-septate, hyaline, smooth-walled.

##### Culture characteristics.

Conidia germinated on PDA, producing germ tubes within 10 hours. *Colonies* on PDA reached a diameter of 29 mm after 49 days of incubation at 25 °C, exhibiting an irregular shape with radially furrowed at the centre and velvety surface, white to pale brown in PDA medium.

##### Material examined.

Thailand • Chiangmai, Mushroom Research Center (MRC), on rotting wood in a terrestrial habitat, 11 September 2020, Jing-Yi Zhang, Y251 (MFLU 24–0035, holotype), ex-type living culture MFLUCC 24–0090 = GZCC 24–0149.

**Figure 2.**
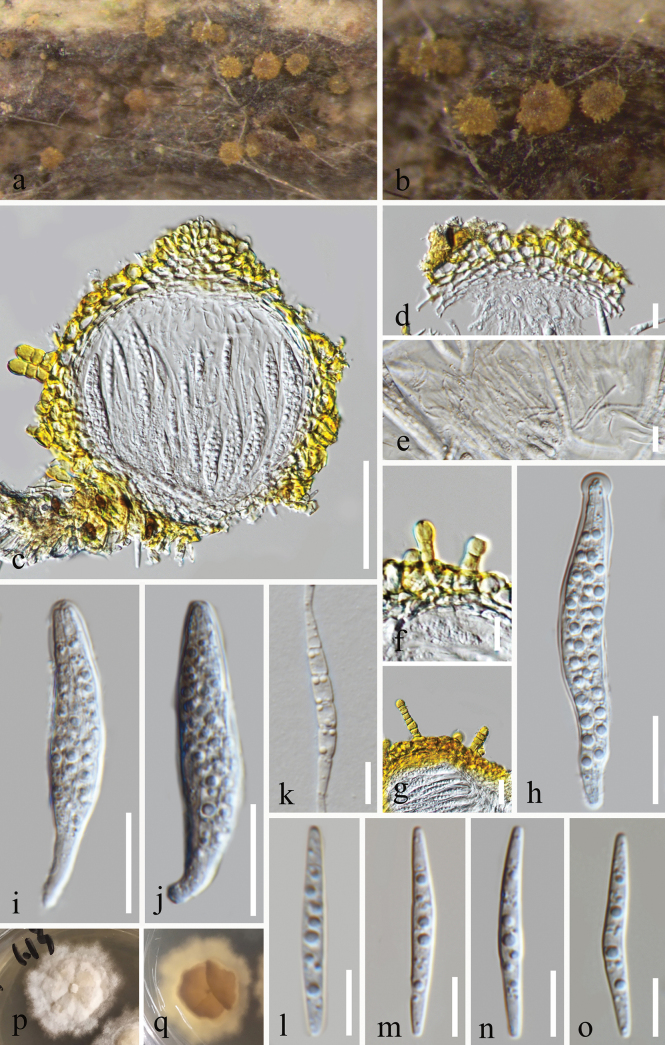
*Helicosporiumrubrum* (MFLU 24–0035, holotype) **a, b** ascomata on the host surface **c** vertical sections of ascomata **d** peridium **e** hamathecium pseudoparaphyses **f, g** short projections of setae-like **h–j** asci **l–o** ascospores **k** germinating ascospore **p, q** surface and reverse colonies on PDA after 49 days of incubation at 25 °C. Scale bars: 50 μm (**c**); 20 μm (**g–j**); 10 μm (**d–f, k–o**).

##### Notes.

Our newly isolated strains (MFLUCC 24–0090 and GZCC 24–0149) formed a sister relationship with *H.flavum* (MFLUCC 16–1230 and GZCC 24–0487), supported by 100% ML/1.00 PP support (Fig. 1). A comparison of the ITS, LSU, and *tef*1-α sequence data between our strain (MFLUCC 24–0090) and *H.flavum* (MFLUCC 16–1230) revealed nucleotide base differences of 19/506 bp (3.8%, including four gaps), 10/757 bp (1.3%, without gap), and 26/904 bp (2.9%, including four gaps), respectively. Morphologically, *Helicosporiumrubrum* resembles *H.flavum* (MFLU 17–0704) in having solitary, scattered, globose to subglobose, bright reddish-yellow to brown-yellow ascomata; bitunicate, fissitunicate, cylindrical to clavate asci; and fusiform, straight to slightly curved, multi-septate, hyaline ascospores (Brahmanage et al. 2017). However, *H.rubrum* differs from *H.flavum* by having smaller asci (51–77 × 8–12.5 µm vs. 70–130 × 12–16 µm) and shorter ascospores (27–35 × 3–4.5 µm vs. 40–60 × 8–12 µm) (Brahmanage et al. 2017). Additionally, *H.flavum* exhibits brown to black-brown setae, absent in *H.rubrum* (Brahmanage et al. 2017). Therefore, based on morphological and molecular data, we propose *Helicosporiumrubrum* as a new species (Chethana et al. 2021).

#### ﻿ Neohelicomyces maolanensis

Taxon classificationFungiTubeufialesTubeufiaceae

J. Ma & Y.Z. Lu sp. nov.

D81D1F4C-2471-5853-93C6-7BF93318FF18

902921

Facesoffungi Number: FoF16909

Fig. 3

##### Holotype.

HKAS 128855.

##### Etymology.

‘‘*maolanensis*’’ refers to its collection site, where the fungus was collected.

##### Description.

***Saprobic*** on decaying wood in a forest. ***Sexual morph*** Undetermined. ***Asexual morph*** Hyphomycetous, helicosporous. ***Colonies*** on natural substrate superficial, effuse, solitary, scattered or gregarious, white to pale brown. ***Mycelium*** partly immersed, partly superficial, composed of pale brown to brown, branched, septate, guttulate, smooth. ***Conidiophores*** 201–230 μm long, 3–4.5 μm wide (x̄ = 220 × 3.5 μm, n = 20), macronematous, mononematous, procumbent, solitary, cylindrical, tapering at tip, flexuous, unbranched, septate, slightly constricted at septa, hyaline to pale brown, smooth-walled, thick-walled. ***Conidiogenous cells*** 13.5–18.5 μm long, 2.5–4 μm wide (x̄ = 16 × 3.5 μm, n = 30), holoblastic, monoblastic to polyblastic, integrated, intercalary or terminal, cylindrical or subcylindrical, with a denticulate protrusion, truncate at apex after conidial secession, hyaline to pale brown, smooth-walled. ***Conidia*** solitary, acropleurogenous, helicoid, tapering towards the rounded ends, developing on tooth-like protrusions, 13.5–19 μm diam. and conidial filament 2.5–3 μm wide (x̄ = 16 × 2.8 μm, n = 30), 105–134 μm long (x̄ = 116.5 μm, n = 30), aseptate, tightly coiled 3–3^3^/_4_ times, becoming loosely coiled when the conidia are young in water and not becoming loose when the conidia mature in water, guttulate, hyaline, smooth-walled.

##### Culture characteristics.

Conidia germinated on PDA, producing germ tubes within 8 hours. *Colonies* on PDA reached a diameter of 24 mm after 37 days of incubation at 25 °C, exhibiting an irregular shape with a flat surface and undulate margin, pale brown to brown in PDA medium.

**Figure 3.**
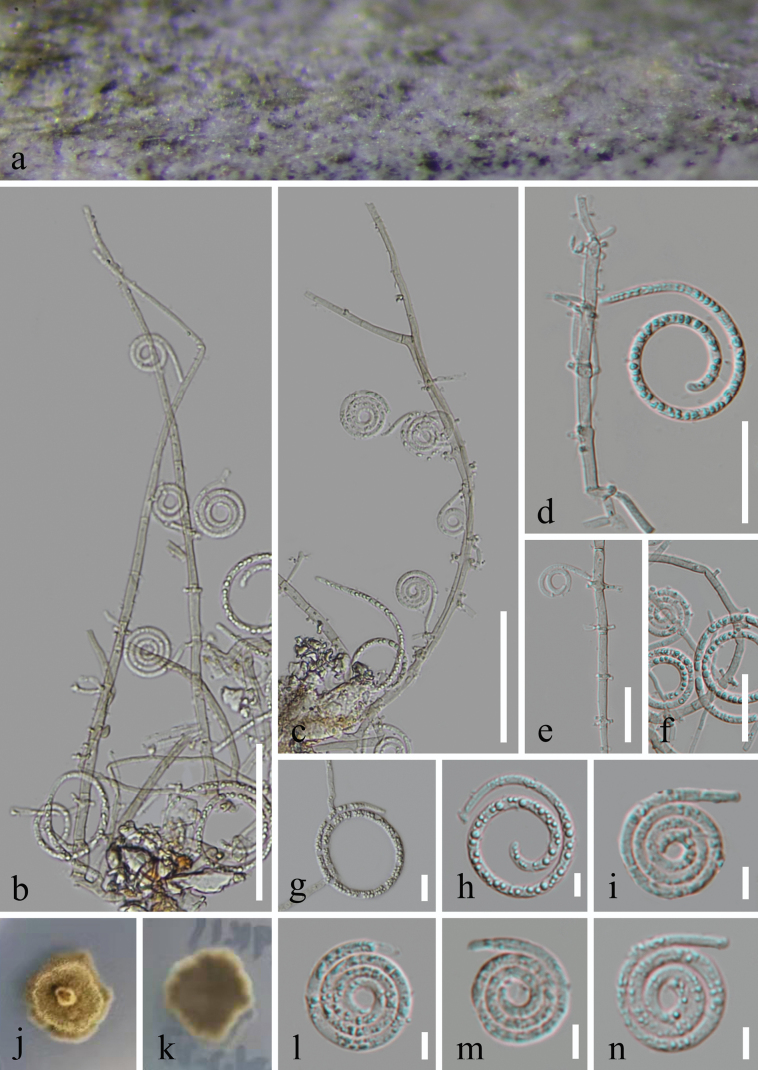
*Neohelicomycesmaolanensis* (HKAS 128855, holotype) **a** colonies on the host surface **b–d** conidiophores, conidiogenous cells with conidia **e, f** conidiogenous cells **h, i, l–n** conidia **g** germinated conidium **j, k** surface and reverse colonies on PDA after 37 days of incubation at 25 °C. Scale bars: 50 μm (**b, c**); 20 μm (**d–f**); 10 μm (**g**); 5 μm (**h, i, l–n**).

##### Material examined.

China • Guizhou Province, Qiannan Buyi and Miao Autonomous Prefecture, Libo County, on rotting wood in a terrestrial habitat, 10 April 2022, Jian Ma, MN5 (HKAS 128855, holotype), ex-type living culture GZCC 23–0079; • Ibid., MN5.1 (GZAAS 23–0634, paratype), ex-paratype living culture GZCC 23–0148.

##### Notes.

*Neohelicomycesmaolanensis* (HKAS 128855) is morphologically similar to *N.deschampsiae* (CBS H–23590) in having erect, flexuous, multi-septate, brown conidiophores; monoblastic to polyblastic, intercalary, pale brown conidiogenous cells; and solitary, hyaline conidia (Crous et al. 2019a). However, *Neohelicomycesmaolanensis* can be distinguished from *N.deschampsiae* by its greater number of coils (3–3^3^/_4_ times vs. 2–3 times), smaller conidial diameter (13.5–19 μm vs. 19–22 μm), and wider conidial filaments (2.5–3 μm vs. 2–2.5 μm) (Crous et al. 2019a). According to the phylogenetic analysis (Fig. 1), our new isolates formed a distinct lineage within the clade, which comprises *N.helicosporous* (GZCC 23–0633 and GZCC 23–0634), *N.hyalosporous* (GZCC 16–0086), *N.qixingyaensis* (CGMCC 3.25569), and *N.subtropicus* (GZCC 23–0076 and GZCC 24–0147), indicating that GZCC 23–0079 and GZCC 23–0148 represent a distinct species. Therefore, we propose *Neohelicomycesmaolanensis* (GZCC 23–0079 and GZCC 23–0148) as a novel species based on molecular and morphological evidence.

#### ﻿ Neohelicomyces subtropicus

Taxon classificationFungiTubeufialesTubeufiaceae

J. Ma & Y.Z. Lu sp. nov.

3AECE0E3-1CB6-506C-ABE1-2BD401CF2587

902922

Facesoffungi Number: FoF16910

Fig. 4

##### Holotype.

HKAS 128847.

##### Etymology.

‘‘*subtropicus*’’ named after the climate from which the holotype was found.

##### Description.

***Saprobic*** on decaying wood in a forest. ***Sexual morph*** Undetermined. ***Asexual morph*** Hyphomycetous, helicosporous. ***Colonies*** on natural substrate superficial, effuse, solitary, gregarious, white to pale brown. ***Mycelium*** partly immersed, partly superficial, composed of pale brown to brown, branched, septate, guttulate, smooth. ***Conidiophores*** up to 420 μm long, 2.5–5.5 μm wide (x̄ = 3.5 μm, n = 30), macronematous, mononematous, erect, solitary or in a group, cylindrical, long or short, tapering at tip, flexuous, mostly branched, septate, slightly constricted at septa, hyaline to pale brown at base, becoming hyaline toward apex, smooth-walled, thick-walled. ***Conidiogenous cells*** 10.5–19.5 μm long, 2–5.5 μm wide (x̄ = 15.5 × 3.5 μm, n = 35), holoblastic, monoblastic to polyblastic, integrated, intercalary or terminal, cylindrical or subcylindrical, with a denticulate protrusion, truncate at apex after conidial secession, hyaline to pale brown, smooth-walled. ***Conidia*** solitary, acropleurogenous, helicoid, dry, tapering towards the rounded ends, developing on tooth-like protrusions, 14.5–16.5 μm diam. and conidial filament 1.5–3 μm wide (x̄ = 15.5 × 2 μm, n = 25), 87–132 μm long (x̄ = 110.5 μm, n = 25), aseptate, tightly coiled 2–2^3^/_4_ times, becoming loosely coiled when the conidia are young in water and not becoming loose when the conidia mature in water, guttulate, hyaline, smooth-walled.

**Figure 4.**
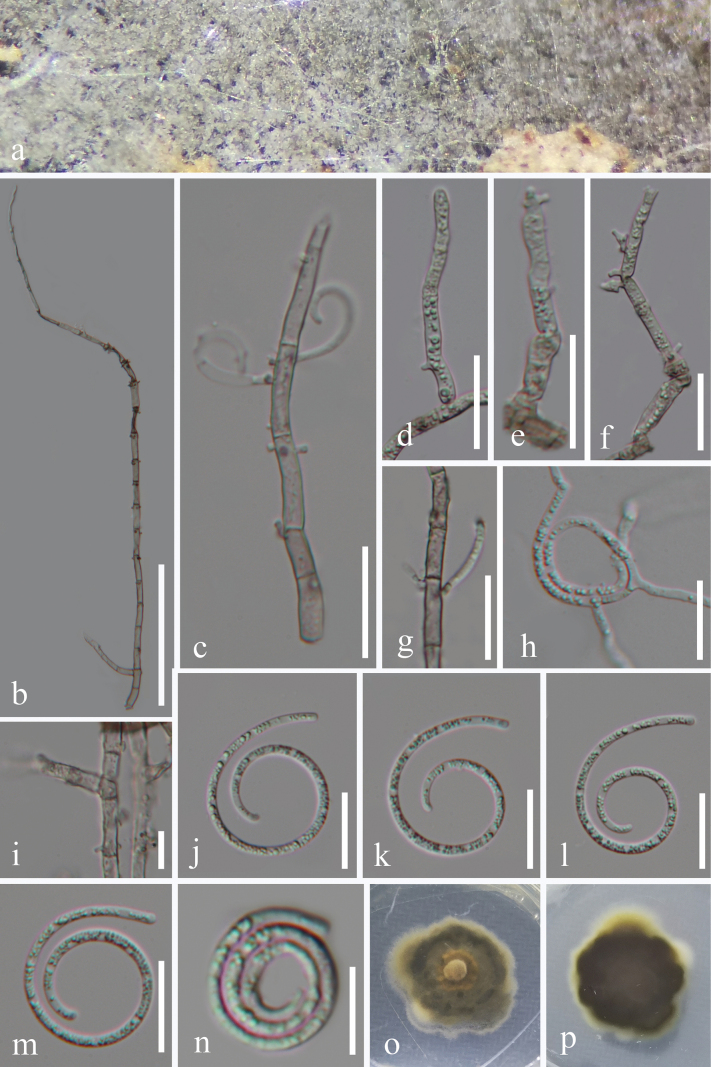
*Neohelicomycessubtropicus* (HKAS 128847, holotype) **a** colonies on the host surface **b–e** conidiophores, conidiogenous cells with conidia **f, g, i** conidiogenous cells **j–n** conidia **h** germinated conidium **o, p** surface and reverse colonies on PDA after 46 days of incubation at 25 °C. Scale bars: 100 μm (**b**); 20 μm (**c–h, j–m**); 10 μm (**i, n**).

##### Culture characteristics.

Conidia germinated on PDA, producing germ tubes within 12 hours. *Colonies* on PDA reached a diameter of 44 mm after 46 days of incubation at 25 °C, exhibiting an irregular shape with a flat surface and undulate margin, pale brown to black brown in PDA medium.

##### Material examined.

China • Guizhou Province, Qiannan Buyi and Miao Autonomous Prefecture, Libo County, on rotting wood in a terrestrial habitat, 10 April 2022, Jian Ma, MN2 (HKAS 128847, holotype), ex-type living culture GZCC 23–0076; • Ibid., MN2.1 (GZAAS 23–0632, paratype), ex-paratype living culture GZCC 23–0147.

##### Notes.

Phylogenetically, our new isolates (GZCC 23–0076 and GZCC 23–0147) formed a sister clade with *N.helicosporus* (GZCC 23–0633 and GZCC 23–0634) and *N.hyalosporus* (GZCC 16–0086) (Fig. 1). A comparison of the ITS, LSU, *tef*1-α, and *rpb*2 sequence data between our strain (GZCC 23–0076) and *N.hyalosporus* (GZCC 16–0086) revealed nucleotide base differences of 29/515 bp (5.6%, including 12 gaps), 1/842 bp (0.1%, without gap), 21/912 bp (2.3%, including one gap), and 22/1045 bp (2.1%, without gap), respectively. Morphologically, our newly collected specimen (HKAS 128847) differs from *N.helicosporus* (HKAS 134923) and *N.hyalosporus* (HKAS 97441) in having longer conidiophores (up to 420 μm vs. 105–199 μm and 210–290 μm, respectively) (Lu et al. 2018b; Ma et al. 2024a). Additionally, *Neohelicomycessubtropicus* exhibits branched conidiophores, which are absent in *N.helicosporus* and *N.hyalosporus* (Lu et al. 2018b; Ma et al. 2024a). Therefore, we propose *Neohelicomycessubtropicus* as a new species based on morphological comparison and multi-gene phylogenetic analysis.

## ﻿Discussion

This study collected five saprobic taxa from terrestrial habitats in China and Thailand. Based on a multi-gene phylogenetic analysis (using LSU, ITS, *tef*1-α, and *rpb*2), combined with morphological descriptions, the sexual species, *Helicosporiumrubrum*, and two new helicosporous *Neohelicomyces* species, *N.maolanensis* and *N.subtropicus*, are proposed.

To date, 286 helicosporous species, including the two new species described in this study, are classified within the family Tubeufiaceae (Tubeufiales, Dothideomycetes, Ascomycota) (Ma et al. 2024b). The family Tubeufiaceae comprises 31 helicosporous genera, *viz. Acanthohelicospora*, *Acanthostigmina*, *Acrohelicosporium*, *Berkleasmium*, *Camporesiomyces*, *Chlamydotubeufia*, *Dematiohelicoma*, *Dematiohelicomyces*, *Dematiohelicosporum*, *Helicangiospora*, *Helicoarctatus*, *Helicodochium*, *Helicohyalinum*, *Helicoma*, *Helicomyces*, *Helicosporium*, *Helicotruncatum*, *Helicotubeufia*, *Hyalohelicoon*, *Hyalohelisphora*, *Hyalotubeufia*, *Neoacanthostigma*, *Neohelicomyces*, *Neohelicosporium*, *Parahelicomyces*, *Pleurohelicosporium*, *Pseudohelicoon*, *Pseudohelicosporium*, *Pseudotubeufia*, *Thaxteriella*, and *Tubeufia* (Liu et al. 2018; Lu et al. 2018a, 2018b, 2023a, 2023b; Lu and Kang 2020; Ma et al. 2023a, 2023b, 2024b; Sruthi et al. 2024). Of which, only 11 genera (*Acanthohelicospora*, *Acanthostigmina*, *Berkleasmium*, *Helicangiospora*, *Helicoma*, *Helicosporium*, *Helicotubeufia*, *Neoacanthostigma*, *Neohelicosporium*, *Thaxteriella*, and *Tubeufia*) have been confirmed to have asexual-sexual links based on molecular data and/or morphological characteristics (Samuels et al. 1979; Boonmee et al. 2011; Hyde et al. 2016; Lu et al. 2017b, 2017c, 2018b, 2023a, 2023b; Liu et al. 2018; Lu and Kang 2020; Ma et al. 2023a, 2023b, 2024b; Sruthi et al. 2024). Moreover, three genera (*Camporesiomyces*, *Chlamydotubeufia*, and *Parahelicomyces*) have documented sexual morphs, although their asexual morphs remain undescribed (Hyde et al. 2017, 2020; Hsieh et al. 2021).

Previous multi-gene phylogenetic analyses have demonstrated that the sexual and asexual morphs of certain helicosporous genera exhibit significant diversity (Lu et al. 2017b, 2018b; Boonmee et al. 2014; Ma et al. 2023b, 2024b). For instance, the sexual morph of *Tubeufialatispora* is characterized by black ascomata with attached unbranched, dark brown setae, sessile asci, and fusiform to cylindrical ascospores, while *T.javanica* possesses cream-white ascomata, short pedicellate asci, and filiform ascospores. Conidial morphology among *Tubeufia* species also shows diversity. Most *Tubeufia* species have hyaline, coiled conidia (Lu et al. 2017b, 2018b; Ma et al. 2024b), but *T.africana* has ellipsoid to ovoid, spherical to obclavate conidia (Kuo and Goh 2021; Ma et al. 2024b), *T.dictyospora* has globose to subglobose, ovoid to irregular conidia (Lu et al. 2018b), and *T.muriformis* features dorsoventrally curved, muriform conidia (Ma et al. 2023b). Additionally, *T.sessilis* has muriform, curved, coiled conidia (Ma et al. 2024b), and *T.subrenispora* produces dorsoventrally curved to subreniform, multicelled muriform dark brown conidia (Zhao et al. 2006, 2007; Ma et al. 2024b). Some *Tubeufia* species also bear small, globose secondary conidia (Zhao et al. 2006, 2007; Ma et al. 2024b). Therefore, multi-gene phylogenetic analyses are essential for correctly identifying these taxa.

Morphological similarities between helicosporous genera can sometimes be misleading, but molecular data often reveal distinct taxonomic positions (Ma et al. 2024b). For example, the asexual morph of *Helicosporium* species closely resembles *Acanthohelicosporascopula* and *Pseudohelicosporiumlaxisporum* in their pale yellow to yellow-green colonies, setiferous, brown to dark brown conidiophores, and helicoid, hyaline to yellow-green conidia (Linder 1929; Zhao et al. 2007; Boonmee et al. 2014; Brahmanage et al. 2017; Lu et al. 2017a, 2018b, 2022; Xiao et al. 2023; Ma et al. 2024b). However, multi-gene phylogenetic analysis shows that *Acanthohelicospora*, *Helicosporium*, and *Pseudohelicosporium* are distributed across different genera within the family Tubeufiaceae (Ma et al. 2024b).

## Supplementary Material

XML Treatment for Helicosporium rubrum

XML Treatment for Neohelicomyces maolanensis

XML Treatment for Neohelicomyces subtropicus

## ﻿Additional information

### Author ORCIDs

Tao Peng https://orcid.org/0009-0005-4690-3277

Yong-Zhong Lu https://orcid.org/0000-0002-1033-5782

Song Bai https://orcid.org/0000-0002-1972-2834

Jing-Yi Zhang https://orcid.org/0000-0003-0606-6169

Xing-Juan Xiao https://orcid.org/0009-0003-8769-4534

Na Wu https://orcid.org/0000-0002-4837-9019

Jian Ma https://orcid.org/0009-0008-1291-640X

### Data availability

All of the data that support the findings of this study are available in the main text.
